# Detection Method for Bolt Loosening Based on Summation Coefficient of Absolute Spectrum Ratio

**DOI:** 10.3390/s25010246

**Published:** 2025-01-04

**Authors:** Haoyang Guo, Jianfeng Zhong, Bin Feng, Yulong Chen, Shuncong Zhong

**Affiliations:** Fujian Provincial Key Laboratory of Terahertz Functional Devices and Intelligent Sensing, School of Mechanical Engineering and Automation, Fuzhou University, Fuzhou 350108, China; 220220031@fzu.edu.cn (H.G.); 220227062@fzu.edu.cn (B.F.); 220227054@fzu.edu.cn (Y.C.); sczhong@fzu.edu.cn (S.Z.)

**Keywords:** Summation Coefficient of Absolute Spectrum Ratio, chirp Fourier transform, reference spectrum curve, detection index for bolt loosening degree, early stage of bolt loosening

## Abstract

A bolt loosening detection method based on the summation coefficient of the absolute spectrum ratio technique is proposed to address the prevalent issue of bolt loosening in mechanical connections. This proposed method involves initially collecting vibration and rotation speed signals of the motor bolt connection structure, acquiring the baseline spectrum curve of a healthy structure and the spectrum curves of non-healthy structures under different degrees of bolt looseness through chirp Fourier transform (CFT). Subsequently, the spectrum ratio curves between healthy and non-healthy structures are calculated for different degrees of bolt loosening, and then the Summation Coefficient of Absolute Spectrum Ratio (SCASR) is defined to indicate the looseness. In the mathematical model, a linear relationship is observed between the SCASR and the frequency shift of the resonance peak. To standardize the results of bolt loosening detection, the SCASR could be divided by the number of points in the fixed frequency band to obtain the average of SCASR as the detection index for bolt loosening. Finally, a linear fitting equation is established between the bolt torque and the average of SCASR, so that the change in the average value can be used to determine whether the bolt is loose and evaluate the severity of bolt looseness. The detection performance of this proposed method has been effectively validated through both simulation and experiments. Experimental results indicate that the proposed method can effectively detect bolt loosening, particularly in its early stages, using low-frequency band data in three axes.

## 1. Introduction

The bolted joint structure boasts advantages such as low cost, ease of installation and disassembly, and robust load-bearing capacity. It is precisely due to these merits that bolted connections find widespread application across various domains including civil engineering, mechanical engineering, and aerospace. However, due to the harsh working conditions that bolted joints often face, they may endure prolonged exposure to vibration, shock, and fatigue caused by alternating loads, leading to loosening or even detachment of the bolted connection structure. In severe cases, it may even cause damage to buildings or equipment. In engineering structures, if the loosening status of bolt connections cannot be promptly detected in their early stages and corresponding measures cannot be taken, it will not only increase the cost of later operation and maintenance of mechanical equipment but may even lead to structural damage, causing serious safety accidents [1].

In recent years, a lot of scholars have conducted extensive research on the detection of loosening in bolted connection structures. Among these, three main methods for detecting bolt loosening have emerged: methods based on guided wave technique [2,3,4,5,6,7], vibration technique [8,9], and piezoelectric impedance [10,11,12,13]. These three methods are widely utilized for the identification and monitoring of bolt loosening. As artificial intelligence (AI) and image processing technologies continue to advance, numerous researchers have endeavored to integrate these with the three primary methodologies previously discussed, as well as with acoustic-based detection techniques [14], forming another major method for bolt loosening detection based on artificial intelligence [15,16].

The bolt loosening detection method based on vibration technology is essentially the reduction in bolt connection stiffness, which leads to changes in vibration information after structural loosening and damage. Bolt loosening can be detected based on changes in structural dynamic parameters or vibration response characteristics caused by loosening and damage. Wu et al. [17] utilized the average vibration response energy as an indicator of bolt loosening damage, constructing a damage index based on the relative change in this indicator before and after bolt loosening. The position of the loosened bolt is determined according to the magnitude of the damage index. Wang et al. [18] proposed a bolt-loosening identification method based on the Short-Time Fourier Transform (STFT) and Image Pixel (IP) time-frequency features. Through experimental validation, they demonstrated that the STFT-IP indicator effectively characterizes different pre-tightening states of bolted connection structures. Li et al. [19] proposed a method for diagnosing bolt loosening faults by extracting time-domain indicator features and employing Principal Component Analysis (PCA). Successful validation of the method was obtained on a motor-based bolt loosening test rig. Cao et al. [20] utilized the Short-Time Fourier Transform method to extract vibration response characteristics under high-temperature environments. They combined the fundamental frequency, the amplitude of the second harmonic, and the ratio of the second harmonic to the fundamental frequency of composite material connectors to comprehensively assess the loosening status of the connection structure. Experimental results demonstrated that the proposed method evaluates the loosening characteristics of composite material bolted connections in high-temperature vibration environments. Xie et al. [21] proposed a bolt loosening detection method based on the combination of Variational Mode Decomposition (VMD) and time-frequency sensitive features with a Least Squares Support Vector Machine (LSSVM), targeting four different states of bolt loosening. F. Huda et al. [22] proposed a vibration testing and health monitoring system based on an impulse response excited by laser ablation to detect bolted joint loosening. The effectiveness of the present approach is verified by simulations and experimental results, which are able to detect and identify loose bolt positions in a six-bolt joint cantilever.

This study proposes a method for detecting bolt loosening severity based on the Summation Coefficient of Absolute Spectrum Ratio (SCASR). The methodology begins by collecting vibration and rotational speed signals from the healthy bolted connection structure under sweep frequency excitation, using the resulting spectrum curve as a reference. Then, the spectrum curves of bolted connection structures with different degrees of loosening under the same sweep frequency excitation are obtained and analyzed by calculating the ratios with the reference curve. This process yields the average of the Summation Coefficient of Absolute Spectrum Ratio (SCASR), which serves as an indicator for detecting the severity of bolt loosening. Finally, a linear fitting equation is established between bolt torque and the loosening detection indicator to evaluate detection performance.

The objective of this study is to identify an indicator that intuitively reflects varying degrees of bolt loosening, including early-stage looseness. Compared to existing vibration-based techniques for detecting bolt loosening, this study offers the following innovations and advantages:

1. A novel SCASR-based loosening detection indicator is proposed, providing an intuitive and effective measurement of bolt loosening severity, particularly excelling in early-stage detection.
2. The method enables precise detection of bolt loosening severity using vibration and rotational speed data in the low-frequency range, facilitating a significantly more streamlined detection of complex bolted connection structures in motor systems.
3. This method demonstrates excellent detection performance across vibration data in all three axes, further validating its applicability and reliability in multi-axial detection scenarios.

## 2. Impact of Bolt Loosening on Structural Natural Frequencies

### 2.1. Bolt Loosening Detection Principle and Simulation Verification

The motor is connected to the foundation via bolts, resulting in a contact interface between the motor and the foundation. Generally, the stiffness of the contact interface is a crucial component of the overall stiffness of the mechanical structure. In the force-closed kinematic chain formed by the interaction between the motor and the foundation, the motor and foundation can be regarded as rigid bodies with high intrinsic stiffness, while the contact interface exhibits lower stiffness. Consequently, the stiffness of the contact interface plays a dominant role in determining the overall structural stiffness [23,24]. As the stiffness of the contact interface increases, the natural frequency of the mechanical structure rises. Conversely, as the damping of the contact interface increases, the natural frequency of the mechanical structure decreases [25].

#### 2.1.1. Calculation of Stiffness and Damping in Bolt Connections

**Figure 1:**
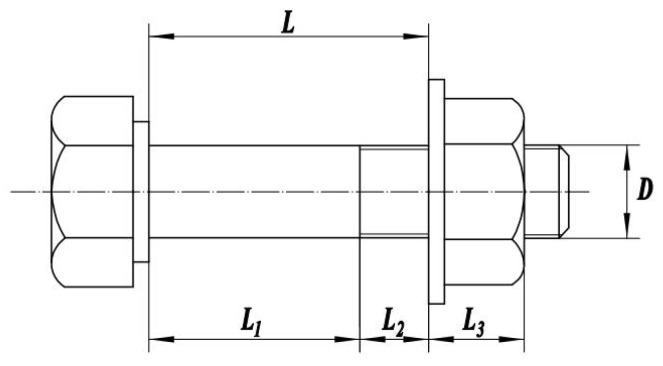
The bolt dimensional diagram.

The axial stiffness of the bolt is closely related to the elastic modulus of the bolt material and its geometric dimensions. A schematic diagram of the bolt’s geometric dimensions is shown in Figure 1.

The empirical formula for calculating the axial stiffness of a bolt is as follows:

(1) $$K_{L}=\frac{E_{L}AA_{s}}{A_{s}L_{1}+A\left(L_{2}+L_{3}\right)}$$

where $E_{L}$ is the elastic modulus of the bolt material; A is the cross-sectional area of the unthreaded shank of the bolt; $L_{1}$ is the length of the bolt shank; $L_{2}$ is the length of the threaded portion between the bolt head and the nut; $L_{3}$ is the length of the nut; and $A_{s}$ is the cross-sectional area used for thread strength calculations.

The expression for the cross-sectional area used in thread strength calculations is as follows:

(2) $$A_{s}=\frac{\pi }{4}{\left(\frac{d_{1}+d_{2}}{2}\right)}^{2}$$

where $d_{1}$ is the pitch diameter of the external thread; $d_{2}$ is the thread calculation diameter.

Under external loads, the contact interface between the motor and the foundation exhibits nonlinear damping, which results in stiffness softening. This interface damping significantly impacts the vibration characteristics of the motor-foundation system. Therefore, when analyzing the effect of damping characteristics on the motor-foundation system, it is essential to account for the influence of interface damping.

**Figure 2:**
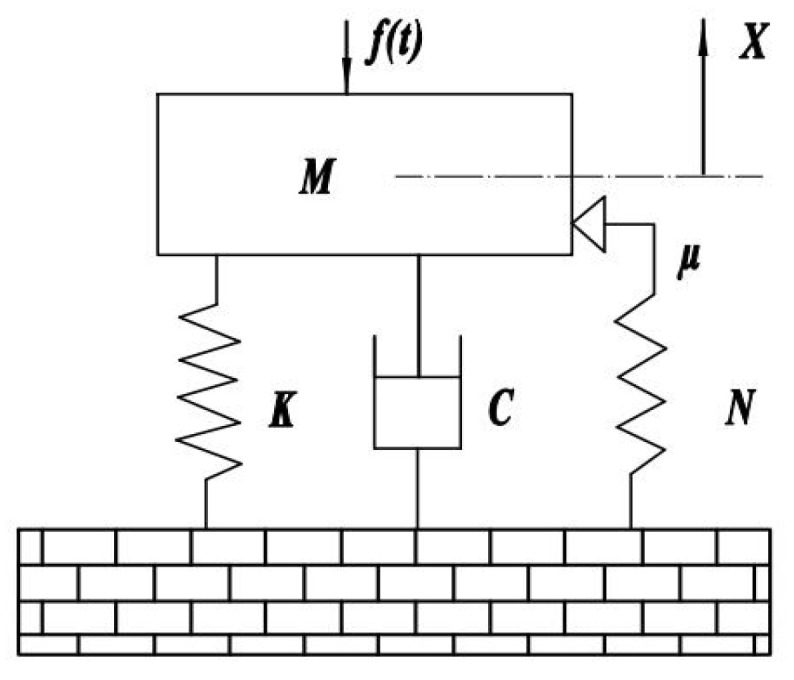
The axial vibration model of a jointed bolt.

As shown in Figure 2, when the bolted connection is subjected to excitation, its displacement is assumed to be:

(3) X=Asinωt

where A is the displacement, which is less than the absolute displacement of the pre-compression.

Where $f\left(t\right)$ is the applied force; M is the mass of the component; K is the total stiffness of the interface connection; C is the viscous damping coefficient of the interface; μ is the coefficient of dry friction (typically set to 0.3); and N is the normal contact force at the contact surface.

The calculation expression for the normal contact pressure can be expressed as:

(4) $$N=N_{T}+N_{P}$$

where $N_{T}$ is the peak-to-peak tangential normal contact pressure of the interface; $N_{P}$ is the peak-to-valley tangential normal contact pressure of the interface.

The total damping of the bolted connection is:

(5) $$f_{d}=f_{\xi }+f_{c}=2\xi \sqrt{\left(1+\lambda \right)}\omega_{c}\omega A\cos\omega t+\frac{N_{T}}{N}\mu \left[X_{0}-A\sin\omega t\right]\omega_{c}^{2}sign\left(x\right)$$

where $f_{\xi }$ is the viscous damping of the connecting bolt; $f_{c}$ is the dry damping of the contact interface; λ is the ratio of bolt tensile stiffness to interface compressive stiffness; and $sign\left(x\right)$ is the sign function; $\xi =\frac{C}{2M\omega_{0}}$.

#### 2.1.2. Motor Vibration Equation

When studying motor vibration issues, the motor can be considered as a mechanical system. Each mechanical system has its own inherent properties, including degrees of freedom, mass, stiffness, and damping matrices. Factors such as shaft imbalance, poor bearing assembly quality, unstable installation foundation, and the influence of electromagnetic forces inside the motor can all affect the vibration response of the mechanical system. Based on the classical vibration equation, the motor vibration equation can be expressed as follows:

(6) $$M\ddot{u}\left(t\right)+C\dot{u}\left(t\right)+Ku\left(t\right)=F\left(t\right)$$

where A is the displacement, which is less than the absolute displacement of the pre-compression.

Here, M represents the n×n mass matrix of the system; C denotes the n×n damping matrix of the system; K represents the n×n stiffness matrix of the system; $\ddot{u}\left(t\right)$ is the n-order vector of acceleration responses of the particles; $\dot{u}\left(t\right)$ is the n-order vector of velocity responses of the particles; $u\left(t\right)$ represents the n-order vector of displacement responses of the particles; and $F\left(t\right)$ is the excitation from the vibration source.

Once the motor system is determined, the mass matrix and stiffness matrix of the system are also established and are typically obtained through theoretical calculations. For the damping matrix of the system, linear damping is considered, meaning the damping is linearly related to the system’s mass and stiffness. Thus, the damping matrix can be expressed as [26]:

(7) $$\left[C\right]=a\left[M\right]+b\left[K\right]$$

#### 2.1.3. Mechanical Model of the Motor Foundation

**Figure 3:**
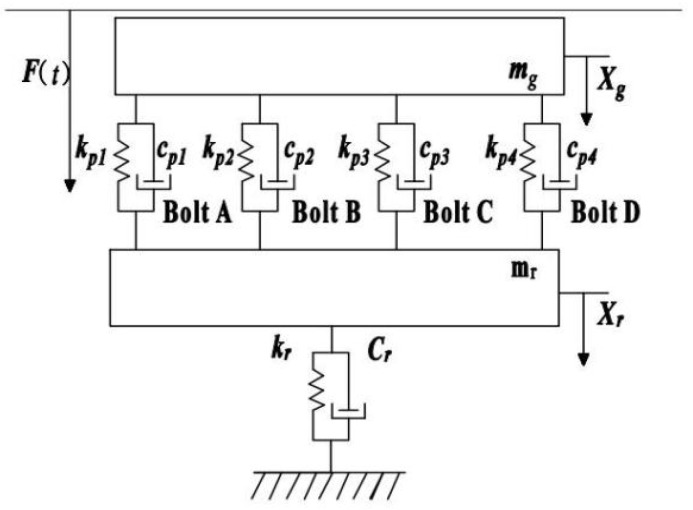
The mechanical model of the motor and foundation.

Common motors typically consist of substructures such as the shaft, bearings, bearing housing, and casing. When the motor is in operation, the vibration energy from the rotor is transmitted sequentially through these substructures and eventually to the foundation via the connecting bolts. Based on the mechanical vibration transmission relationships, a mechanical model of the motor-foundation system is constructed, as shown in Figure 3.

Assuming that the motor vibration is transmitted to the foundation through four connecting bolts, the vibration dynamics equation of the motor-foundation system can be expressed as follows:

(8) $$M\ddot{x}\left(t\right)+C\dot{x}\left(t\right)+Kx\left(t\right)=F\left(t\right)$$

where: $M=\left[\begin{array}{cc} M_{g} & 0\\ 0 & M_{r} \end{array}\right]$; $C=\left[\begin{array}{cc} C_{g}+C_{p} & -C_{p}\\ -C_{p} & C_{r}+C_{p} \end{array}\right]$; $K=\left[\begin{array}{cc} K_{g}+K_{p} & -K_{p}\\ -K_{p} & K_{r}+K_{p} \end{array}\right]$; $x=\left[\begin{array}{c} x_{g}\\ x_{r} \end{array}\right]$; $c_{p}=c_{p1}+c_{p2}+c_{p3}+c_{p4}$; $k_{p}=k_{p1}+k_{p2}+k_{p3}+k_{p4}$.

The vibration characteristic parameters of the connected components in the system include stiffness and damping parameters [27], which can be expressed as $a=\left(c_{p1},c_{p2},c_{p3},c_{p4},k_{p1},k_{p2},k_{p3},k_{p4}\right)$. The dynamic characteristics of the joint vary depending on the preload of the bolts. In other words, different degrees of bolt looseness result in varying connection characteristics between the motor frame and the foundation, which in turn affects the vibration response of the foundation [28]. By introducing a bolt connection characteristic vector, the dynamic equation of the motor-foundation system, including the connection characteristic vector for loose bolts, is described as follows:

(9) $$M\ddot{x}\left(t\right)+C\left(a\right)\dot{x}\left(t\right)+K\left(a\right)x\left(t\right)=F\left(t\right)$$

Thus, solving Equation (9) yields the displacement response vector:

(10) $$x=A\sin\left(n\omega t+u\right)\;\left(n=1,2,3,\cdots \right)$$

where, $A\left(a\right)$ is the complex displacement vector, which is related to the bolt connection characteristic vector a and the inherent properties of the motor-foundation system. u represents the phase lag of the displacement response. Equation (10) indicates that variations in the degree of bolt looseness lead to corresponding changes in the stiffness and damping characteristics at the interface between the motor and the foundation. Consequently, changes in the connection characteristic vector a result in alterations to both the complex displacement vector $A\left(a\right)$ and the overall natural frequencies of the connected structure. Due to nonlinear factors such as contact, friction, and preload in the bolt connections, it is extremely complex to analyze this problem accurately through theoretical methods alone. Therefore, current research often combines experimental data for investigation [29].

#### 2.1.4. Simulation of the Experimental Multi-Bolt Connection System

**Figure 4:**
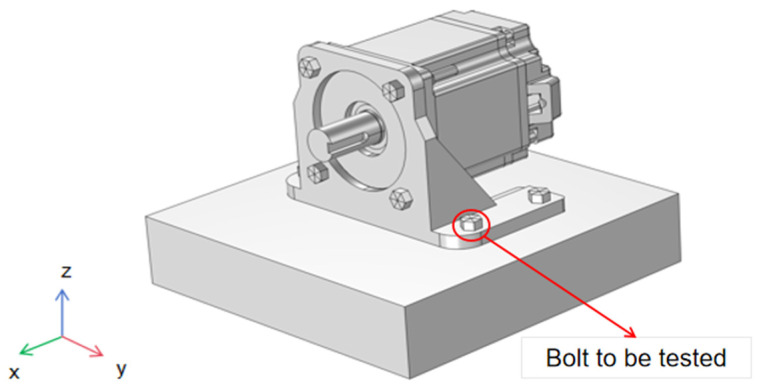
Schematic diagram of simulation model for multi-bolt connection structure in experimental setup.

**Figure 5:**
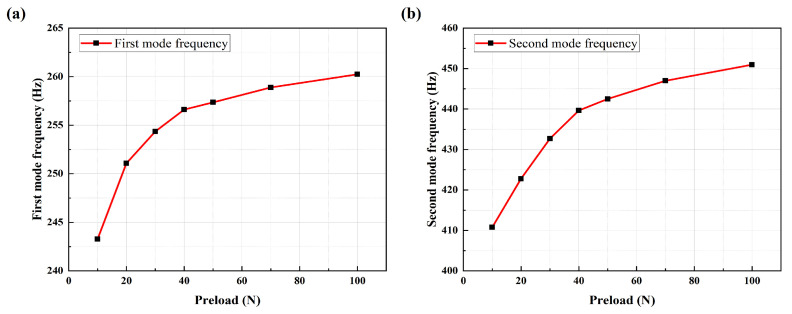
The line chart of (**a**) the first mode natural frequencies and (**b**) second mode natural frequencies of the multi-bolt connection system under different preload conditions.

The above equations indicate that bolt loosening alters the bolt preload, leading to changes in the stiffness of the bolted joint structure, which in turn causes variations in the structural resonance frequency. Therefore, it is necessary to determine whether similar changes occur in actual complex bolted joint structures using finite element analysis. Using COMSOL Multiphysics 6.2 software, a simulation model of the experimental multi-bolt connection structure was established, as shown in Figure 4. Four M6 × 16 inner hexagonal head bolts were used to secure the servo motor and the bracket structure to the fixed platform. Conduct a modal analysis of the motor structure presented in this paper using COMSOL, incorporating the effect of bolt preload through the “Bolt Preloading” module. The specific simulation settings are as follows: set the interface between the bolts, the motor base, and the mounting platform as an “identity boundary pair”, and configure the interface between the motor base and the mounting platform as “contact pairs”. The contact method is set to “penalty function”, with a friction coefficient of 0.3. A hexahedral mesh is applied to the bolt structure and the mounting platform, while a tetrahedral mesh is used for the remaining parts. The maximum mesh element size is set to 0.002. Additionally, the motor base is defined with a fixed constraint. Finally, in the study, the analysis steps “Bolt Preloading” and “Eigenfrequency” are run sequentially to obtain the modal frequencies of the motor structure under bolt preload, as illustrated in Table 1 and Figure 5.

The simulation primarily investigates the impact of the pre-stress force of the fastening bolts on the servo motor bracket on the natural frequency of the overall structure. Due to the complexity of the electronic components inside the servo motor, modeling is time-consuming and not the focus of the calculation. Therefore, finite element discretization in non-critical areas does not require detailed specifications. The internal structure of the servo motor could be simplified, using counterweights to ensure that the mass and inertia of the servo motor remain consistent.

From Figure 5 and Table 1, it can be observed that as the preload increases, both the first-mode and second-mode frequencies of the structure also increase. However, the rate of increase gradually diminishes, and when approaching the maximum preload, the mode frequencies exhibit almost no further increase. Due to bolt loosening, the first four modal frequencies exhibit a leftward shift in the spectral peak frequency, indicating that as the mode order increases, the frequency shift becomes more pronounced with changes in preload. This also verifies the validity of the experimental system in detecting the degree of bolt loosening.

### 2.2. Principle of Detection Method Based on Summation Coefficient of Absolute Spectrum Ratio

The above analysis demonstrates that in bolted joint structures, the resonance frequency changes with variations in preload, causing the spectrum curve, i.e., the amplitude-frequency curve to shift accordingly. However, relying solely on the shift of the spectrum curve to identify changes in resonance frequency can be challenging due to the influence of data noise and the quality of the spectrum curve. Additionally, when the degree of bolt loosening is small, it can be difficult to detect changes in resonance frequency. Therefore, the use of the summation coefficient of the spectrum ratio has been proposed to enhance the sensitivity in detecting the degree of bolt loosening.

To explain the phenomenon of an increase in the average value of the spectrum ratio due to leftward peak frequency offset within a certain range on a mathematical level, a simple Gaussian distribution model was taken into consideration. Assuming that both the reference spectrum curve and the offset spectrum curve can be approximated by Gaussian functions. It assumes that the vibration signal exhibits a single, complete resonance peak within a specific frequency range. This is a common assumption in practice, as many physical processes produce spectrum peaks that approximate Gaussian distributions. The Gaussian function for the reference spectrum curve is given by

(11) $$A_{0}\left(f\right)=A_{0,\max}\exp\left(-\frac{{\left(f-f_{0}\right)}^{2}}{2\sigma^{2}}\right)+b$$

**Figure 6:**
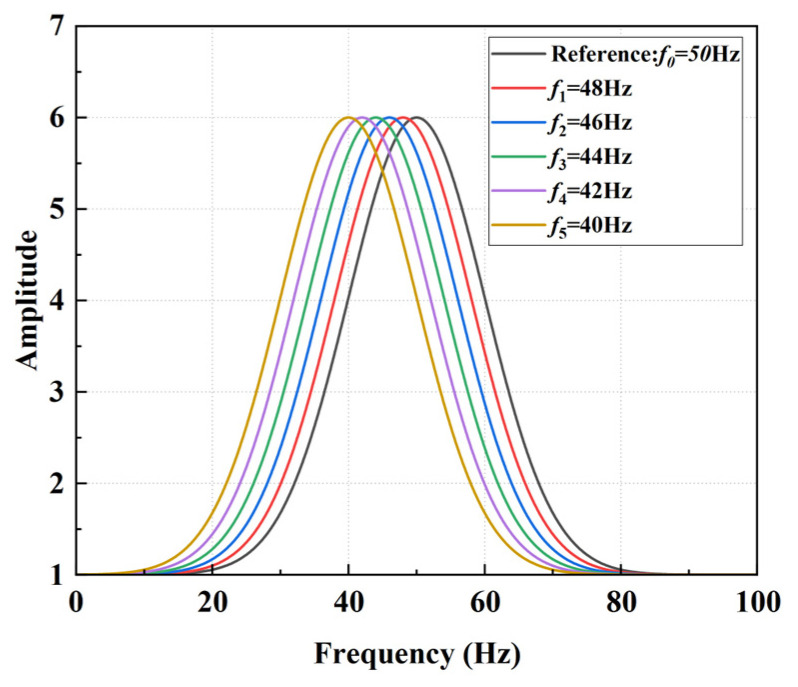
Gaussian function with uniform peak variation in the range of 40–50 Hz.

The Gaussian function for the spectrum curve after leftward shift is given by

(12) $$A\left(f\right)=A_{\max}\exp\left(-\frac{{\left(f-\left(f_{0}-\Delta f\right)\right)}^{2}}{2\sigma^{2}}\right)+b$$

where $A_{0,\max}$ and $A_{\max}$ are the maximum amplitudes of the reference and shifted spectrum curves, respectively, $f_{0}$ is the peak frequency of the reference spectrum curve, Δf is the offset of the peak frequency, σ is the standard deviation of the Gaussian function, controlling the width of the peak, and $A\left(f\right)$ and $A_{0}\left(f\right)$ as illustrated in Figure 6.

The sum of the ratios, denoted as *S*, can be expressed as

(13) $$S=\sum \frac{A\left(f\right)}{A_{0}\left(f\right)}$$

Substituting the Gaussian functions

(14) $$S=\sum \frac{A_{\max}\exp\left(-\frac{{\left(f-\left(f_{0}-\Delta f\right)\right)}^{2}}{2\sigma^{2}}\right)+b}{A_{0,\max}\exp\left(-\frac{{\left(f-f_{0}\right)}^{2}}{2\sigma^{2}}\right)+b}$$

Due to the possibility of $A_{0,\max}$ and $A_{\max}$ being similar or equal, and since *b* is small, the expression for the sum of the ratios *S* can be simplified as

(15) $$S=\sum \exp\left(\frac{{\left(f-f_{0}\right)}^{2}-{\left(f-\left(f_{0}-\Delta f\right)\right)}^{2}}{2\sigma^{2}}\right)$$

The sum *S* of the ratios can be approximated using an integral form over a certain frequency range (such as $f_{1}$ to $f_{2}$)

(16) $$S\approx \int_{f_{1}}^{f_{2}}\exp\left(\frac{\Delta f\left(f_{0}-f-0.5\Delta f\right)}{\sigma^{2}}\right)df$$

**Figure 7:**
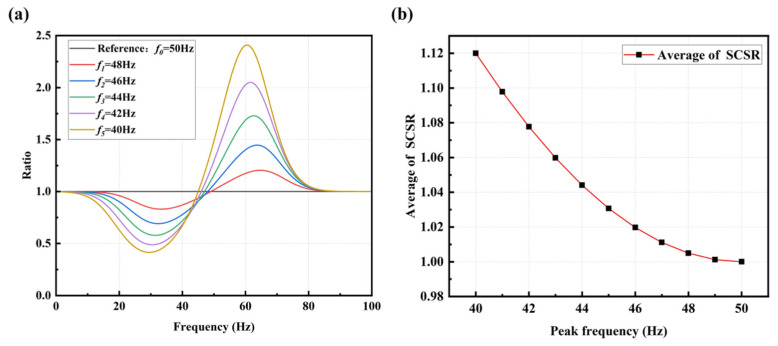
(**a**) Spectrum ratio curves at different peak frequencies; (**b**) Averaged SCSR versus offset of peak frequency.

On the left side of the intersection $f=f_{0}-\Delta f/2$ between the shifted spectrum curve and the reference one, the ratio between them varies between 0 and 1. On the right side of the intersection $f=f_{0}-\Delta f/2$, when Δf>1, the ratio between the reference spectrum curve and the shifted spectrum curve significantly increases. This increase in magnitude is markedly greater than the decrease observed on the left side, as depicted in Figure 7a.

Therefore, by summing up the ratios of the amplitude values corresponding to each shifted spectrum curve to the reference one, the sum of the spectrum ratios for each shifted spectrum curve is obtained. Each shifted spectrum curve corresponds to a different bolt loosening condition, so the summation coefficient of the spectrum ratio curve, i.e., the Summation Coefficient of Spectrum Ratio (SCSR) for each bolt connection condition under a torque of *x* N·m is

(17) $$S\left(x\right)=\sum_{i=f_{1}}^{f_{2}}\frac{A\left[x_{health}\left(i\right)\right]}{A\left[x\left(i\right)\right]}$$

where $\left[f_{1},f_{2}\right]$ represents the selected frequency range, and $A\left[x\left(i\right)\right]$ is the amplitude value of the *i*-th point on the spectrum ratio curve under *x* N·m.

To standardize and compare the changes in bolt loosening degree, the average value $s\left(x\right)$ of SCSR is computed. It allows the relationship curve between the shifted peak frequency and the average of SCSR to be obtained, as shown in Figure 7b.

(18) $$s\left(x\right)=\frac{\sum_{i=f_{1}}^{f_{2}}\frac{A\left[x_{health}\left(i\right)\right]}{A\left[x\left(i\right)\right]}}{f_{2}-f_{1}}$$

**Figure 8:**
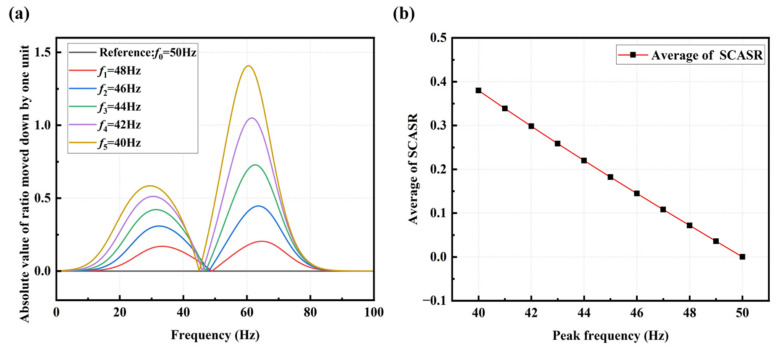
(**a**) Absolute of spectrum ratio curves at different peak frequencies; (**b**) Averaged SCASR versus the offset of peak frequency.

From the graph, it can be observed that the averaged SCSR increases exponentially with a decrease in the peak frequency. However, for early-stage bolt loosening (corresponding to small peak frequency offsets), the change in this index is minimal, making it difficult to observe the loosening status accurately. To amplify the trend of the index changes and enhance the detection effectiveness of early-stage loosening, the entire ratio curve is shifted down by one unit and then taken the absolute value, resulting in the Summation Coefficient of Absolute Spectrum Ratio (SCASR), as shown in Figure 8a. Finally, the average value of SCASR is computed, and this average value is taken as the bolt loosening detection index. Thus, the relationship curve between the averaged SCASR and the shifted peak frequency is obtained, as shown in Figure 8b. It can be observed that they exhibit a linear relationship, allowing for clear observation of index changes even for small peak frequency offsets.

**Figure 9:**
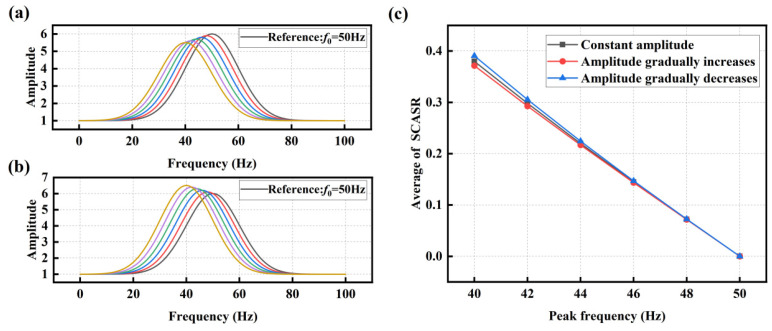
(**a**) Gaussian function where the amplitude gradually decreases with the shift of the peak frequency; (**b**) Gaussian function where the amplitude gradually increases with the shift of the peak frequency; and (**c**) The relationship curves between the averaged SCASR and the offset of peak frequency under conditions where the amplitude gradually increases, decreases, or remains constant with the shift of the peak frequency.

Considering the practical experimental conditions, where amplitude fluctuations often occur, such as the cases of gradual decrease and gradual increase in amplitude shown in Figure 9a,b. The averaged SCASR shown in Figure 9c for these two cases reveals that the curve still demonstrates a reasonably good linearity.

**Figure 10:**
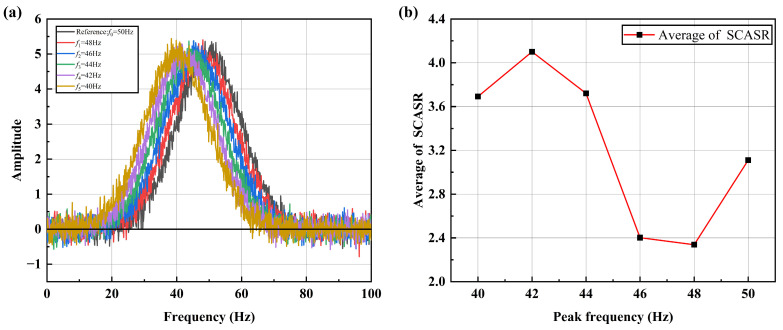
(**a**) Spectrum curves at different peak frequencies under noise; (**b**) Averaged SCASR versus the offset of peak frequency under noise.

**Figure 11:**
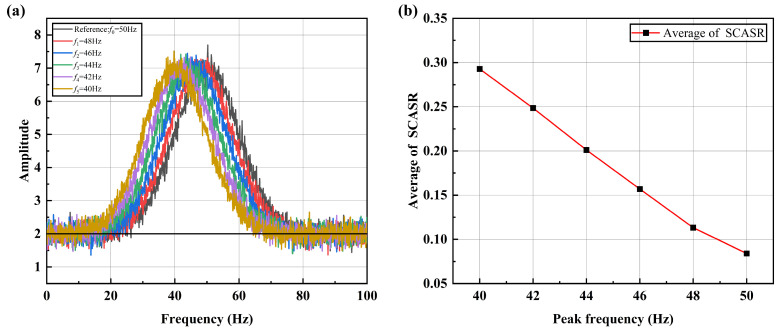
(**a**) Spectrum curves after upward translation at different peak frequencies under noise; (**b**) Averaged SCASR versus the offset of peak frequency under noise.

However, due to the presence of significant noise components in actual vibration signals, it is necessary to further discuss the impact of noise on this indicator. Here, Gaussian noise is added to each frequency shift curve, as shown in Figure 10a. It can be observed in Figure 10b that the ratio variation becomes irregular. This is because the ratio is highly sensitive to the small values caused by noise. By simultaneously shifting all frequency shift curves upward by two, as shown in Figure 11a, it can be observed that the ratio curve restores its linear relationship in Figure 11b. Here, Gaussian noise with a standard deviation of $\sigma_{n}=0.2$ is added to each frequency shift curve, as shown in Figure 11a. The signal-to-noise ratio (SNR) is calculated as approximately 22.04 dB, indicating the relative strength of the signal compared to the added noise.

**Figure 12:**
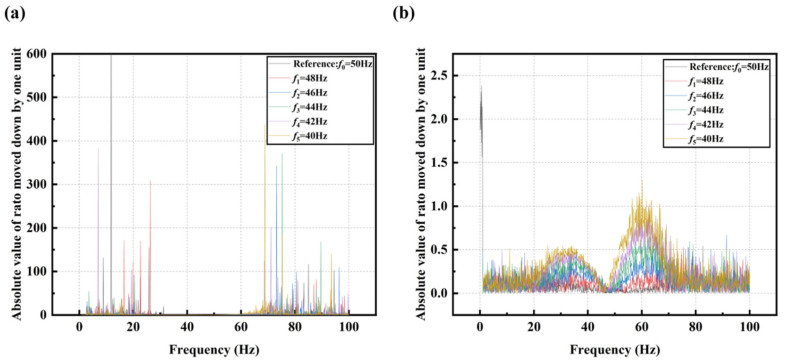
(**a**) Absolute of spectrum ratio curves at different peak frequencies under noise; (**b**) Absolute of spectrum ratio curves after upward translation at different peak frequencies under noise.

From the ratio curves shown in Figure 12, it can be observed that the upward shift of the curves effectively suppresses the impact of noise. In Figure 12a, the amplitude values in the range of 0–1 are highly susceptible to noise interference, leading to distortion in the ratio and obscuring the variation patterns. However, in Figure 12b, where the curves are shifted upward by two units, the variation patterns of the ratios become distinctly observable. Therefore, employing the method of upward shifting the curves is essential for mitigating noise effects in frequency shift detection based on the average of the summed ratios and their variations.

### 2.3. Simulation Validation of Detection Method in Simple Bolted Joint Structure

**Figure 13:**
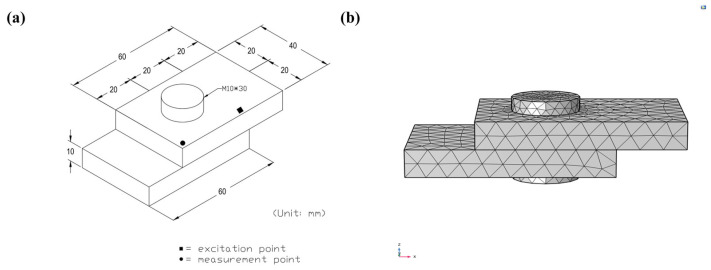
(**a**) Dimensional diagram of the simple bolt connection structure; (**b**) Simulation model of the simple bolt connection structure.

The dimensions of a simple bolt structure are shown in Figure 13a. Using the finite element analysis software COMSOL, a simulation model was established as shown in Figure 13b. The built-in pre-stress analysis module of COMSOL was used to set the pre-stress. An excitation point and a probe point were placed near the bolt to input pulse excitation and obtain vibration signals in the z-direction. The material is set as stainless steel, and contact friction is defined between the two connecting plates.

The pre-stress was set to 10,000 N, 9000 N, 7500 N, 6000 N, 5000 N, and 2500 N, respectively. According to Equation (19), the corresponding bolt torques for these pre-stress values are 20 N·m, 18 N·m, 15 N·m, 12 N·m, 10 N·m, and 5 N·m, respectively.

(19) $$F=\frac{T}{K\times d}$$

where *F*, *T*, *K*, and *d* are the pretension force, the tightening torque, the torque coefficient, and the nominal diameter of the bolt, respectively.

**Figure 14:**
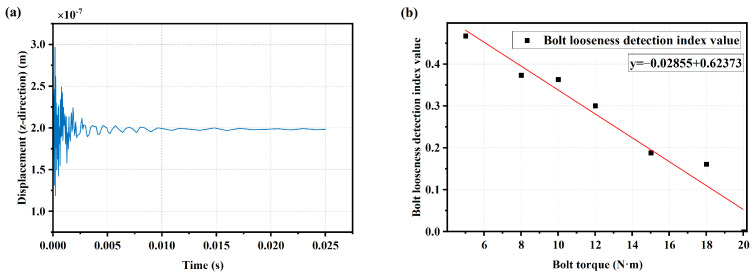
(**a**) Structural response signals under pulse excitation of the healthy structure; (**b**) Linearly regression equation between bolt torque and average of the SCASR.

The condition with a bolt torque of 20 N·m was set as the healthy structure condition. Pulse excitation was applied to the simple bolt connection structure under different bolt pre-stress levels, and the structural response signals under a bolt torque of 20 N·m are shown in Figure 14a. Obtain the spectrum curves of the vibration response signals under the same impulse excitation with different bolt pre-stress levels using FFT. Using the bolt loosening detection method based on the average value of the SCASR of the healthy structure, a linear fitting equation was obtained between the bolt torque and the average of SCASR, as shown in Figure 14b. It can be observed that there is a roughly linear relationship between the bolt torque and the average of SCASR, further validating the performance of using the average of SCASR as an indicator for detecting bolt loosening.

### 2.4. Spectrum Curve Analysis Based on Chirp Fourier Transform

#### 2.4.1. The Principle of the Chirp Fourier Transform

The above simulation validation used FFT to detect the degree of bolt loosening. However, for some rotating machinery, the normal operating frequency range is often set relatively low to avoid the structure’s resonance frequency range, making it difficult to identify the resonance frequency. In such cases, using FFT becomes less appropriate. Therefore, a time-frequency analysis method named chirp Fourier transform (CFT) was employed to calculate the time-frequency spectrum of the vibration signal. The short-time higher-order CFT with the adaptation to the spectral harmonic component *Hn* could be calculated using the expression as follows [30]:

(20) $$X_{Hni}(f_{Hn})=\frac{1}{T_{I}}\int_{0}^{T_{I}}W_{I}(t)x_{i}(t)\exp(-j\Omega_{Hn}(t))dt$$

(21) $$\Omega_{Hn}(t)=\int_{0}^{t}f_{Hn}(\tau )d\tau$$

where *x_i_*(*t*) is the vibration data for the signal segment *i*, *T_I_* is the length of the vibration signal windowed by an internal window *W_I_*(*t*), $\Omega_{Hn}(t)$ is the instantaneous phase of the spectral harmonic component *Hn* that could be calculated as expression (18), $f_{Hn}(\tau )$ is the time dependency of the frequency for the spectral harmonic component *Hn* for which the adaptation will be achieved within the transform. In this study, phase dependence can be estimated from the servo motor speed signal collected synchronously with the vibration signal. During the calculation of CFT, the internal window duration decides the frequency resolution of the spectrum that could be expressed as

(22) $$F_{res}=\frac{1}{T_{I}}$$

The frequency resolution increases with the increase in the period of internal window. To find the resonance peak frequency band of rotating mechanical structures and thereby better observe the impact of bolt tightness on the characteristic frequency of the bolted connection structure, different harmonic of the rotation speed *F*(*Hn*) should be employed, which could be calculated as

(23) $$F(Hn)=Hn*f_{v}(t)$$

where *Hn* is the harmonic order, and *f_v_*(*t*) is the rotation speed of the driving shaft.

#### 2.4.2. Spectrum Analysis for Vibration Data Using Linear-Frequency Excitation

**Figure 15:**
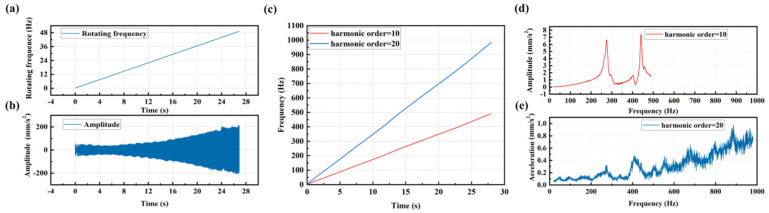
(**a**) Linearly increasing rotating frequency curve; (**b**) Vibration signal excited by the linearly increasing rotating speed; (**c**) Frequency curves for 10th and 20th harmonic orders of the linear increasing rotation frequency curve; (**d**) Spectrum curve for harmonic order of 10 by using CFT; (**e**) Spectrum curve for harmonic order of 20 by using CFT.

To validate the effectiveness of CFT in analyzing high-frequency spectrum curves from vibration data under low-frequency excitation, and to identify the frequency band where the resonance occurs and select the appropriate order, multiple sets of synchronously measured vibration and rotational frequency data of a servo motor under linearly increasing IRS from 0 Hz/min to 50 Hz/min were used for order analysis as shown in Figure 15a,b. By inputting the rotation frequency and the synchronous vibration data into the CFT, spectrum curves for different orders including the resonance peaks could be obtained. To obtain the spectrum for a higher frequency range, harmonic orders of 10 and 20 were selected for the calculation of a higher-order spectrum. The frequency ranges for the 10th and 20th orders are 0–500 Hz and 0–1000 Hz, respectively, as shown in Figure 15c. From the spectrum curves for the 10th and 20th orders shown in Figure 15d,e, the 10th harmonic order spectrum curve provided the best interpolation smoothing effect, with the two resonance peaks in the frequency band appearing relatively smooth and complete. Therefore, the 10th harmonic order spectrum curve can be selected for frequency shift analysis.

## 3. Experiments and Discussions

### 3.1. Description of the Experimental Setup

**Figure 16:**
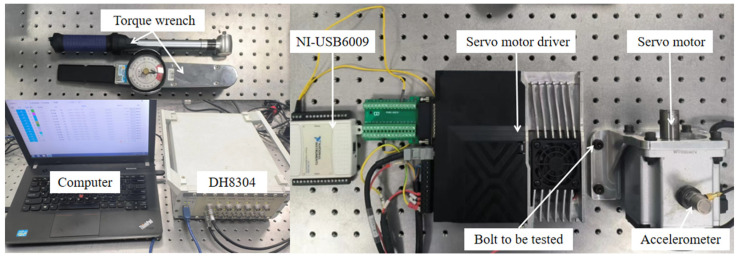
Bolt loosening detection experimental platform.

As shown in Figure 16, the servo motor is fixed on the motor bracket, and then the bracket is fastened to the vibration isolation platform using four internal hexagon M6 × 16 bolts. The bolts are tightened using a torque wrench to ensure secure fastening of the motor with a known torque. The motor shaft rotates horizontally during operation. An accelerometer sensor (Brüel & Kjær (B&K), Nærum, Denmark: 4383V: Reference Sensitivity: 3.16 ± 2% pC/ms^−2^; Measuring Range: Max. operational shock: ±5000 g peak; Frequency Range: (±10% limit): 0.1 to 8400 Hz; Max. Transverse Sensitivity: <4%; Mounted Resonance Frequency: 28 kHz) is placed on the top surface of the servo motor to capture vibrations in the vertical direction. A USB DAQ device (Emerson, Austin, TX, USA: USB6009) is employed to output linearly increasing voltage to the servo motor driver. The rotating speed of the shaft is proportional to input voltage, which acts as an internal excitation of the whole structure. During the excitation, both the excitation voltage and the vibration of the motor are acquired simultaneously using the data acquisition system (DONGHUATEST, Changsha, China: DH8034).

### 3.2. Experimental Analysis for Detecting Different Levels of Bolt Loosening

**Figure 17:**
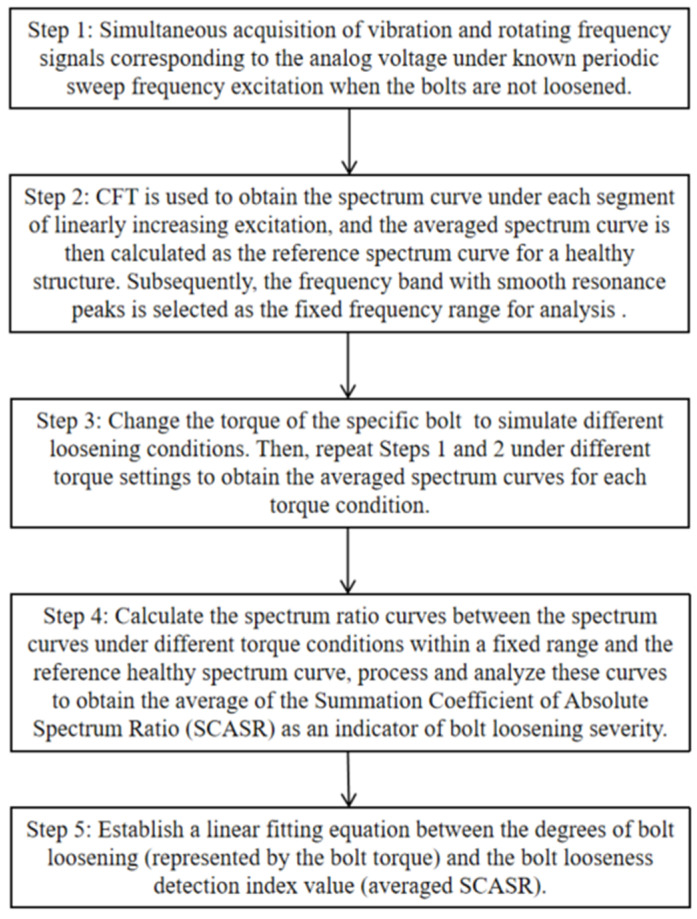
Experimental flowchart for bolt loosening detection.

The general steps of the experiment are as follows in Figure 17.

**Figure 18:**
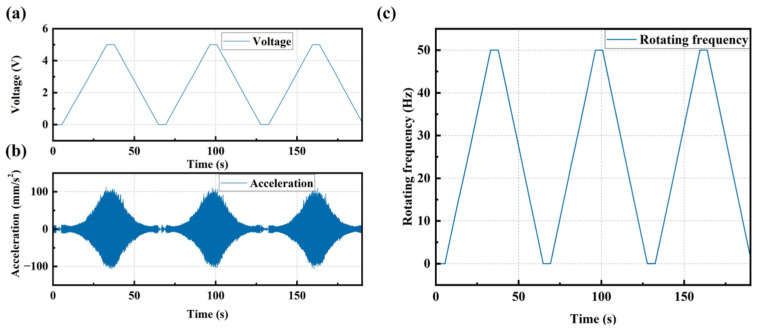
(**a**) Periodic linearly increasing analog voltage curve; (**b**) Vibration signal excited by the linearly increasing rotating speed; (**c**) Linearly increasing rotating frequency curve calculated from the voltage curve.

Step 1: Simultaneous acquisition of vibration and rotating frequency signals corresponding to the analog voltage under known periodic sweep frequency excitation (0–3000 rpm with a constant slope) when the bolts are not loosened, as shown in Figure 18. Figure 18a is a periodic linearly increasing analog voltage curve. Figure 18b is a vibration signal excited by the periodic linearly increasing rotating speed. Figure 18c is a periodic linearly increasing rotating frequency curve calculated from the voltage curve in Figure 18a.

**Figure 19:**
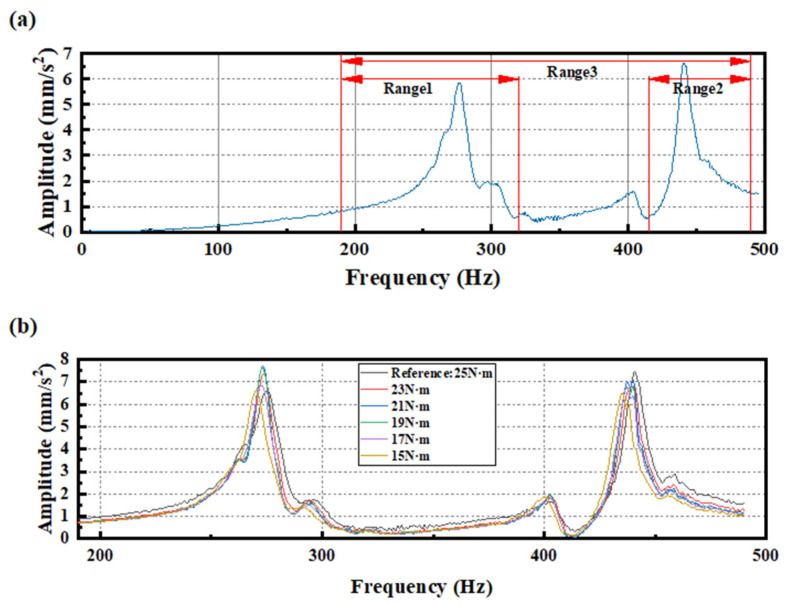
(**a**) Reference spectrum curve of healthy structure; (**b**) Spectrum curves under different degrees of bolt loosening (from 25 N·m to 15 N·m) within a fixed frequency range of 190–490 Hz.

Step 2: CFT is used to obtain the spectrum curve under each segment of linearly increasing excitation, and the averaged spectrum curve is then calculated as the reference spectrum curve for a healthy structure. Subsequently, the frequency band with smooth resonance peaks is selected as the fixed frequency range for analysis (the ranges of 190 to 320 Hz, 420 to 490 Hz, and 190 to 490 Hz were chosen as the three analysis frequency ranges), as shown in Figure 19a.

Step 3: Change the torque of the specific bolt (from 25 N·m to 1 N·m with an interval of 2 N·m, totaling 14 conditions, including the condition without bolts) to simulate different loosening conditions. Then, repeat Steps 1 and 2 under different torque settings to obtain the averaged spectrum curves for each torque condition, as shown in Figure 19b.

Step 4: Calculate the spectrum ratio curves between the frequency spectrum curves under different torque conditions and the reference healthy spectrum curve (all spectrum curves were uniformly shifted upwards by 5 mm/s^2^ before calculating the ratio curves). The resulting spectrum ratio curves were then uniformly shifted downwards by one, and the absolute spectrum ratio curves were taken. Then, the SCASR obtained from the absolute values was divided by the total number of points within the corresponding fixed frequency range to obtain the averaged SCASR, which serves as the index for detecting the degree of bolt loosening.

Step 5: Establish a linear fitting equation between the degrees of bolt loosening (represented by the bolt torque) and the bolt looseness detection index value (averaged SCASR).

**Figure 20:**
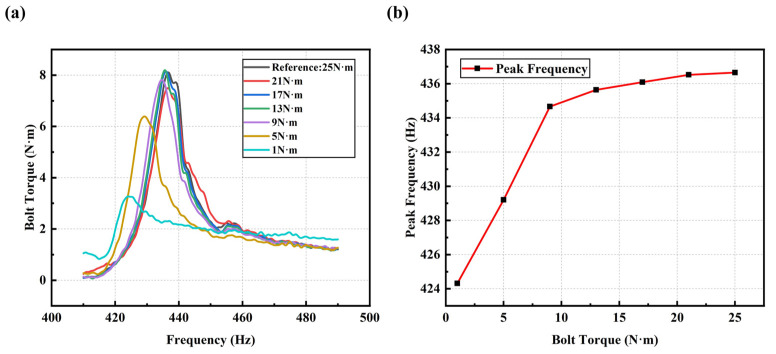
(**a**) Spectrum curves under different degrees of bolt loosening (from 25 N·m to 1 N·m) within a fixed frequency range of 410–490 Hz; (**b**) Second-order eigenfrequencies under different degrees of bolt loosening (from 25 N·m to 1 N·m).

After obtaining the complete experimental data following the aforementioned experimental steps, the peak frequencies extracted from the spectra at different bolt loosening degrees are compared with the simulated data of the motor platform system described in Section 2.1. The second-order eigenfrequency spectrum and the peak frequency variation diagram are shown in Figure 20. It can be observed that as the bolt torque approaches the healthy torque, the variation in the peak frequency decreases. Furthermore, the eigenfrequency is consistent with the simulation results in Section 2.1 of this paper, further validating the rationality of the simulation model.

### 3.3. Analysis of Detection Performance at Different Frequency Ranges

**Figure 21:**
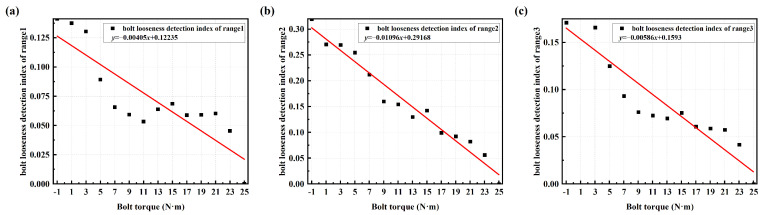
Linear regression equation between bolt torque and bolt looseness detection index for frequency range 1 of 190–290 Hz (**a**); range 2 of 420–490 Hz (**b**); and range 3 of 190–490 Hz (**c**). The bolt torque of −1 N·m represents no bolt.

The linear fitting results of the bolt looseness detection index value (averaged SCASR) with respect to bolt torque for the three analysis frequency ranges are shown in Figure 21a–c. It can be clearly observed that the linear fitting effect is best for analysis frequency range 2, followed by range 3, with range 1 having the poorest effect. From the spectrum curve, it is evident that the resonance peak within analysis frequency range 2 is not only smoother but also exhibits a more pronounced frequency shift. Therefore, the principle for selecting the frequency band is to choose the range within the overall frequency spectrum that contains relatively smoother and higher-order resonance peaks.

In Figure 21b, it is apparent that with the decrease in bolt torque, the bolt looseness detection index value exhibits an increasing trend. Moreover, in the early stages of loosening, i.e., when there is a slight decrease in bolt connection torque, the change in the bolt looseness detection index value is also quite significant. Therefore, the proposed method has good sensitivity for detecting the bolt looseness.

### 3.4. Analysis of Detection Performance on X-Axis and Y-Axis

**Figure 22:**
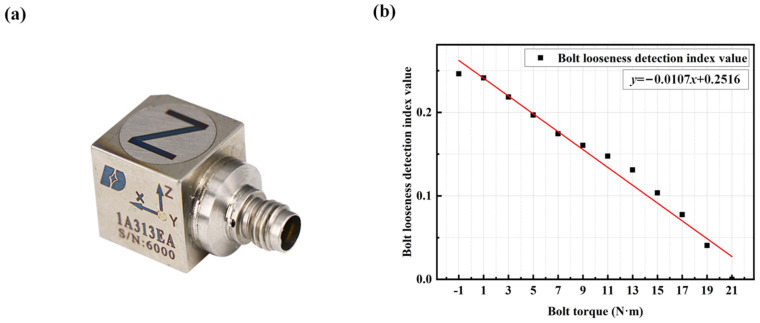
(**a**) Triaxial piezoelectric accelerometer (DONGHUATEST: 1A314E); (**b**) Linear regression equation between bolt torque and bolt looseness detection index in the *Z*-axis direction. The bolt torque of −1 N·m represents no bolt.

To evaluate the general applicability of this detection method, tests were conducted using four M6 × 12 bolts after replacing the uniaxial accelerometer with a triaxial piezoelectric accelerometer (DONGHUATEST: 1A314E: Reference Sensitivity: 10 mV/ms^−2^; Measuring Range: ±50 g; Frequency Range: (±10% limit): 0.5 to 7000 Hz; Max. Transverse Sensitivity: <5%; Mounted Resonance Frequency: >25 kHz), as shown in Figure 22a. The spectrum under the condition of the bolt torque of 21 N·m for all four bolts was used as the reference spectrum for a healthy structure. The previously described method was then applied to process the vibration signals in the X, Y, and Z directions separately. The detection effect in the Z direction had good detection performance in stability and sensitivity as illustrated in Figure 22b. In this section, according to the selection criteria mentioned above, the frequency range of 420–457 Hz is chosen as the fixed frequency band. Subsequently, the detection effects of the other two axes were compared, and the variation patterns in their vibration spectrum were observed.

**Figure 23:**
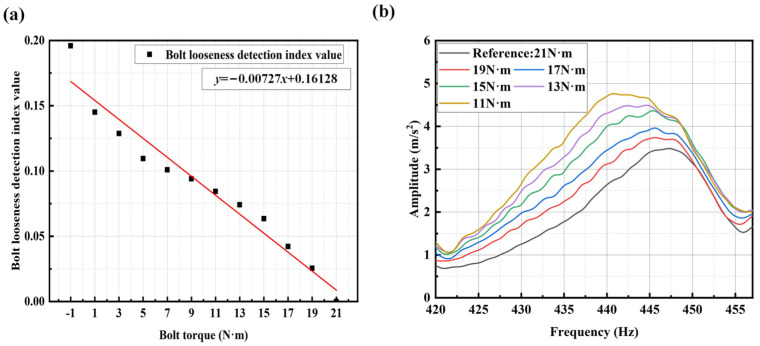
(**a**) Linear regression equation between bolt torque and bolt looseness detection index in the *Y*-axis direction; (**b**) *Y*-axis spectrum within the fixed frequency band under different degrees of bolt loosening (from 21 N·m to 11 N·m). The bolt torque of −1 N·m represents no bolt.

Using the same method to process the vibration data in the *Y*-axis, the relationship curve between the bolt loosening index and bolt torque was obtained, as shown in Figure 23a. It can be observed that the averaged SCASR, as the bolt loosening detection index, exhibits an excellent linear fitting relationship with the bolt torque. From the vibration spectrum of the *Y*-axis in Figure 23b, it is evident that as the bolt torque decreases, the resonance peak frequency gradually shifts to the left, further confirming the effectiveness of the *Y*-axis in detecting bolt loosening. The *Z*-direction and *Y*-direction are in the radial direction of the motor shaft and generally are more sensitive to the change of bolt looseness. Therefore, the detection performance of the proposed method is excellent, even under conditions where there are minor fluctuations in the amplitude of the spectrum.

**Figure 24:**
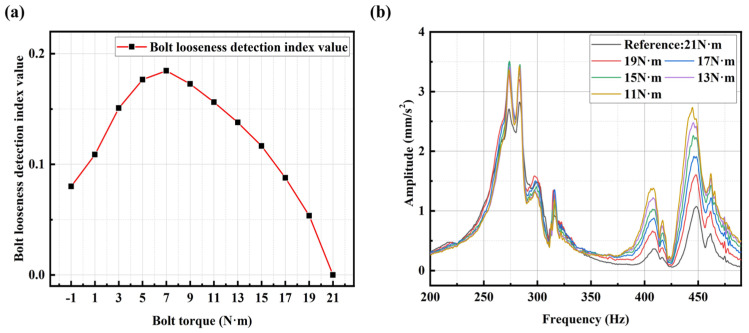
(**a**) The relationship between bolt torque and bolt looseness detection index in the *X*-axis direction; (**b**) *X*-axis spectrum under different degrees of bolt looseness (from 21 N·m to 11 N·m). The bolt torque of −1 N·m represents no bolt.

Continue to use the above method to obtain the relationship curve between bolt torque and bolt looseness index values (averaged SCASR) for the *X*-axis signals, as shown in Figure 24a. It can be observed from the figure that when the bolt torque is 7 N·m or less, there is an abnormal decrease in the bolt loosening detection index value. The cause of the anomaly needs to be identified from the spectrum of the *X*-axis, as shown in Figure 24b. In the early and middle stages of bolt loosening (bolt torque ranges from 21 N·m to 7 N·m), the frequency shift in the *X*-axis spectrum curves is present but not significant. The vibration amplitude along the *X*-axis (the *X*-axis corresponds to the axial direction of the motor) significantly increases during the early and middle stages of bolt loosening due to the effects of centrifugal forces generated by the motor and system nonlinearity, among other factors. Therefore, it can be found that in the early and middle stages of bolt loosening, the bolt looseness detection index (averaged SCASR) still increases with the decrease in the bolt torque which demonstrates a certain level of detection performance.

**Figure 25:**
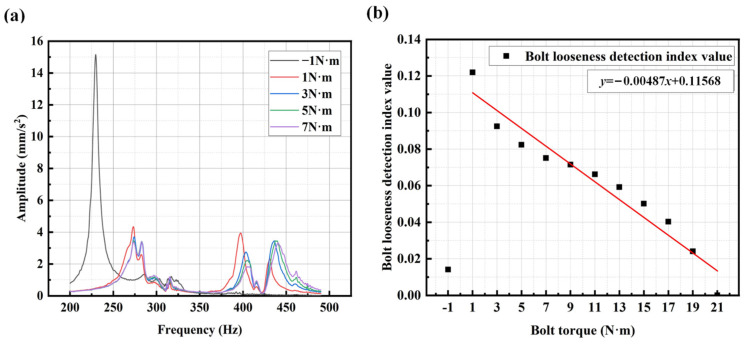
(**a**) *X*-axis spectrum under different degrees of bolt looseness (from 7 N·m to −1 N·m); (**b**) Linear regression equation between bolt torque and the bolt looseness detection index of the 350–420 Hz frequency range in the *X*-axis direction. The bolt torque of −1 N·m represents no bolt.

From the *X*-axis spectrum during the later stages of bolt loosening, as shown in Figure 25a, it is observed that the amplitude in the 420–457 Hz frequency range no longer increases and even begins to decline, which is in stark contrast to the trends observed during the early and middle stages of bolt loosening. Additionally, the significant variation in the *X*-axis vibration amplitude as the degree of bolt loosening increases has a significant impact on the detection performance of the bolt looseness index in this frequency range. Therefore, the frequency range (350–420 Hz) with more stable amplitude variation is used for bolt looseness detection analysis, as shown in Figure 25b. It can be observed from the figure that, except in the case without bolts, the bolt looseness detection index value (averaged SCASR) exhibits a good linear fitting relationship with bolt torque, demonstrating the effectiveness of this method in detecting the degree of bolt loosening on the *X*-axis. Therefore, the frequency range in different directions should be properly selected in real applications.

In summary, the proposed bolt looseness detection index demonstrates good effectiveness in detecting the degree of bolt loosening using vibration signals in the three directions, particularly during the early and middle stages of bolt loosening. Therefore, a vibration signal in any direction under linearly excitation could be used for bolt looseness detection using the proposed method. When high-precision and high-reliability analysis of bolt loosening is required in some applications, the detection indexes (averaged SCASR) in three directions can be employed to comprehensively analyze.

## 4. Conclusions

To address the common challenge of early detection of bolt loosening in mechanical systems, this paper proposes a bolt loosening detection method based on the Summation Coefficient of Absolute Spectrum Ratio (SCASR). First, in the motor-foundation mechanical model, it is demonstrated that bolt loosening significantly affects the characteristic frequencies of the overall connected structure. This is validated through a motor bolt-connection structural model built in COMSOL. Next, a numerical analysis of the SCASR is conducted, revealing that this feature remains highly sensitive to frequency shifts even under conditions of noise and amplitude variations. The feasibility of the proposed method in detecting bolt loosening is further verified using a simplified bolt-connection structure model in COMSOL. Then, to identify the resonance peak frequency band of rotating mechanical structures, this study employs the Chirp Fourier Transform (CFT) to analyze and process vibration and rotational speed data, thereby obtaining the required spectral information within the target frequency range.

Finally, an experimental platform for bolt loosening detection was constructed, where a servo motor applies swept frequency excitation to the bolted joint structure. The results show that this method can effectively detect the degree of bolt loosening in all three axial directions, particularly demonstrating good performance in early-stage loosening detection. The proposed method establishes a research foundation for detecting the degree and location of loosening in multiple-bolt connections.

## Figures and Tables

**Table 1:** The results for the first four mode frequencies under different pre-stress levels.

Preload Force/N	First Mode Frequency/Hz	Second Mode Frequency/Hz	Third Mode Frequency/Hz	Fourth Mode Frequency/Hz
10	243.27	410.77	792.06	1389.2
20	251.08	422.79	809.64	1415.5
30	254.35	432.69	825.20	1445.4
40	256.61	439.64	837.59	1472.1
50	257.35	442.49	843.41	1485.5
70	258.89	446.98	852.41	1506.3
100	260.24	450.95	860.67	1527.2
1000	264.35	458.05	877.17	1578.7
2000	264.87	458.21	877.71	1582.6

## Data Availability

The data presented in this study are available on request from the corresponding author due to the lack of an appropriate database.
